# Studying the Symbiotic Bacterium *Xenorhabdus nematophila* in Individual, Living *Steinernema carpocapsae* Nematodes Using Microfluidic Systems

**DOI:** 10.1128/mSphere.00530-17

**Published:** 2018-01-03

**Authors:** Matthew D. Stilwell, Mengyi Cao, Heidi Goodrich-Blair, Douglas B. Weibel

**Affiliations:** a Department of Biochemistry, University of Wisconsin—Madison, Madison, Wisconsin, USA; b Department of Bacteriology, University of Wisconsin—Madison, Madison, Wisconsin, USA; c Department of Microbiology, University of Tennessee—Knoxville, Knoxville, Tennessee, USA; d Department of Biomedical Engineering, University of Wisconsin—Madison, Madison, Wisconsin, USA; e Department of Chemistry, University of Wisconsin—Madison, Madison, Wisconsin, USA; University of Illinois at Urbana-Champaign

**Keywords:** *Steinernema* nematodes, *Xenorhabdus nematophila*, bacterial single-cell analysis, host-microbe interactions, microfluidics, mutualism, population dynamics, symbiosis

## Abstract

Animal-microbe symbioses are ubiquitous in nature and scientifically important in diverse areas, including ecology, medicine, and agriculture. *Steinernema* nematodes and *Xenorhabdus* bacteria compose an established, successful model system for investigating microbial pathogenesis and mutualism. The bacterium *Xenorhabdus nematophila* is a species-specific mutualist of insect-infecting *Steinernema carpocapsae* nematodes. The bacterium colonizes a specialized intestinal pocket within the infective stage of the nematode, which transports the bacteria between insects that are killed and consumed by the pair for reproduction. Current understanding of the interaction between the infective-stage nematode and its bacterial colonizers is based largely on population-level, snapshot time point studies on these organisms. This limitation arises because investigating temporal dynamics of the bacterium within the nematode is impeded by the difficulty of isolating and maintaining individual living nematodes and tracking colonizing bacterial cells over time. To overcome this challenge, we developed a microfluidic system that enables us to spatially isolate and microscopically observe individual, living *Steinernema* nematodes and monitor the growth and development of the associated *X. nematophila* bacterial communities—starting from a single cell or a few cells—over weeks. Our data demonstrate, to our knowledge, the first direct, temporal, *in vivo* visual analysis of a symbiosis system and the application of this system to reveal continuous dynamics of the symbiont population in the living host animal.

**IMPORTANCE** This paper describes an experimental system for directly investigating population dynamics of a symbiotic bacterium, *Xenorhabdus nematophila*, in its host—the infective stage of the entomopathogenic nematode *Steinernema carpocapsae*. Tracking individual and groups of bacteria in individual host nematodes over days and weeks yielded insight into dynamic growth and topology changes of symbiotic bacterial populations within infective juvenile nematodes. Our approach for studying symbioses between bacteria and nematodes provides a system to investigate long-term host-microbe interactions in individual nematodes and extrapolate the lessons learned to other bacterium-animal interactions.

## Abstract (precis)

This paper describes an experimental system for directly investigating population dynamics of a symbiotic bacterium, *Xenorhabdus nematophila*, in its host—the infective stage of the entomopathogenic nematode *Steinernema carpocapsae*. Tracking individual and groups of bacteria in individual host nematodes over days and weeks yielded insight into dynamic growth and topology changes of symbiotic bacterial populations within infective juvenile nematodes. Our approach for studying symbioses between bacteria and nematodes provides a system to investigate long-term host-microbe interactions in individual nematodes and extrapolate the lessons learned to other bacterium-animal interactions.

## INTRODUCTION

Microbes form symbiotic relationships with organisms in every kingdom of life and in every ecosystem, ranging from mutualism (all partners benefit) to parasitism (some partners benefit, but others are harmed or killed) (1). Many bacterial species are obligate mutualists or obligate pathogens, while others can switch between these two extremes depending on aspects of their external environment, such as host identity (2, 3), abiotic parameters (e.g., temperature) (4–6), or microbial community partners (7). Microbial symbioses are important in a wide and growing range of areas—including medicine and agriculture (8)—as these relationships play a crucial role in host health, development, and nutrition (1, 9). An understanding of the processes underlying the initiation and maintenance of microbial symbioses is important in predicting conditions under which they emerge, as well as strategies to control, prevent, or engineer them. Insights into these processes require surpassing the limitations of traditional microbiology approaches that rely heavily on the *in vitro* growth of microbes under synthetic conditions; techniques that make it possible to study microbes *in situ* within complex and dynamic host environments could have an important impact on the symbiosis field (10, 11). To bypass the logistic, technical, and ethical constraints associated with studying symbiosis in vertebrate mammals, numerous labs have developed model systems centered on invertebrate animals to investigate principles of symbiosis (9, 12).

Invertebrate animals (e.g., nematodes, ants, squid, and coral) and their microbial symbionts provide tractable model systems for studying basic mechanisms and dynamics in host-microbe interactions (9, 13). These model organisms have yielded insights into signaling, recognition, persistence (long-term survival in the host), host development, and nutrient exchange between hosts and symbionts (13). Nematodes are particularly useful model organisms for studying bacterial symbiosis, as they are small, transparent, and relatively simple in terms of multicellular organisms and occupy diverse environmental niches (14). Several bacterium-nematode model systems have been developed to explore basic mechanisms of host-microbe symbiotic interactions, including terrestrial entomopathogenic nematodes associated with gammaproteobacteria; *Laxus oneistus* marine nematodes with surface-colonizing thiotrophic bacteria; and filarial nematodes interacting with their intracellular symbiotic bacterium, *Wolbachia* (11, 15). Recent studies have also explored microbial symbiosis in the model nematode *Caenorhabditis elegans* in the context of recognizing its association with diverse microbes in its natural environment (11, 16).

A well-characterized model of nematode-bacterium symbiosis is the soil-dwelling and entomopathogenic *Steinernema* nematode species and their *Xenorhabdus* bacterial partners. An emerging hypothesis indicates that these mutually beneficial symbionts may have coadapted and coevolved, and studies have revealed molecular determinants that promote transmission and maintenance of their species-specific pairings (17, 18). Among this family of organisms, the *Steinernema carpocapsae* nematode and *Xenorhabdus nematophila* bacterium symbiotic pair and their insect prey together have been established as a tractable system to investigate pathogenesis and mutualism in microbial symbiosis and a relatively simple model to investigate animal-microbe interactions *in vivo* (3, 19, 20).

**FIG 1:**
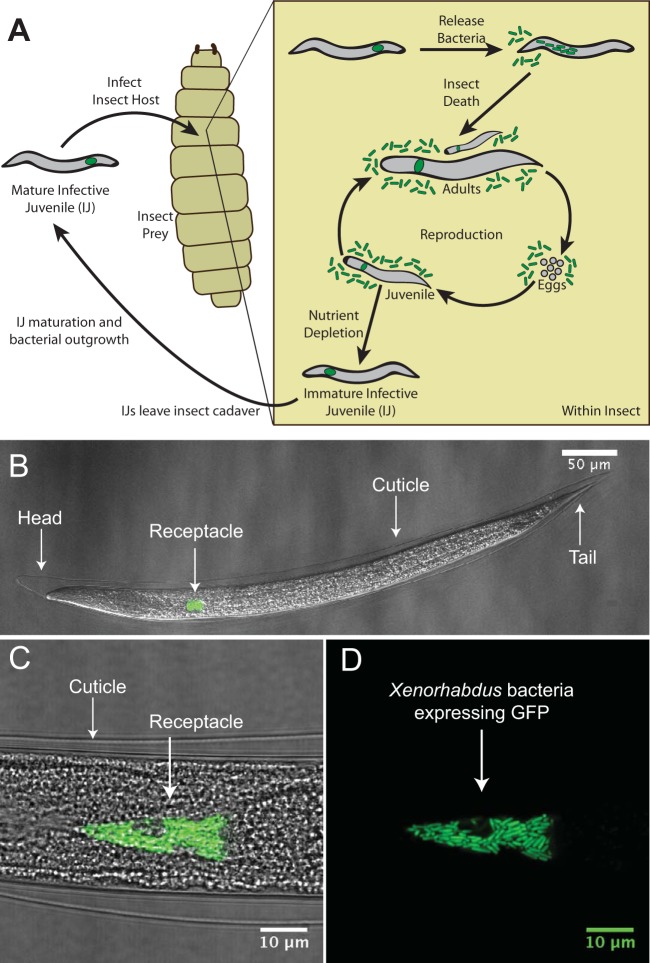
The mutualistic relationship between *Xenorhabdus nematophila* bacteria and *Steinernema carpocapsae* nematodes. (A) Cartoon depicting the tripartite life cycle of *S. carpocapsae* nematodes. Infective juveniles (IJs) infect an insect prey and release *X. nematophila* cells to evade the host immune system and kill the host. Both species use the cadaver’s nutrients for reproduction; upon nutrient depletion, the two organisms reassociate and enter the soil to begin the cycle again. (B to D) Confocal micrographs of the IJ stage of an *S. carpocapsae* nematode carrying GFP-expressing *X. nematophila* cells in the intestinal receptacle. The intravesicular structure (IVS) can be seen in panel D as the dark space below the white arrow and surrounded by bacterial cells.

*X. nematophila* cells occupy an intestinal pocket called the receptacle in the nonfeeding, developmentally arrested stage of nematodes referred to as infective juveniles (IJs) (Fig. 1). As they prey on insects, *S. carpocapsae* IJ nematodes transport bacteria housed in the receptacle, where the bacteria can attach to the intravesicular structure (IVS) (the IVS is seen as the “void” around which the bacteria grow in the top part of Fig. 1D) (21). Upon entering the insect hemocoel (literally “bag of blood”), *S. carpocapsae* nematodes release the population of *X. nematophila*, and together the nematode and bacteria kill the insect and use its nutrients for reproduction. IJ nematodes grow into adults, mate sexually, and produce eggs, which hatch into juvenile nematodes. With sufficient nutrients, juvenile nematodes develop into adults and start the next round of the reproductive cycle (3, 19). A high density of nematodes and depletion of nutrients trigger the formation of pre-IJ nematodes, a transient developmental stage that leads to the formation of colonized IJs (22). Immature IJs leave the cadaver, become mature IJs, and seek a new insect host (23, 24) (Fig. 1).

Entomopathogenic nematodes can be raised and propagated in the laboratory using inexpensive techniques (25). The transparency and hardiness of *S. carpocapsae* nematodes make them amenable to optical microscopy to study the anatomical structures of bacterial localization. This is further facilitated by the genetic tractability of *X. nematophila* and by strains that stably express fluorescent proteins, making them visible within nematodes (Fig. 1) (26). Studies using such tools have revealed discrete stages of bacterial colonization of nematodes in juvenile, adult, pre-IJ, and IJ forms (22) and have demonstrated that these colonization events are species specific, such that only *X. nematophila* and not other *Xenorhabdus* species associates with *S. carpocapsae* nematodes (27). Previous research also revealed that the final population of bacterial cells in the IJ nematode is clonal and that a period of outgrowth occurs in which the bacterial population expands to fill the receptacle (23). In contrast to the smooth exponential growth of *X. nematophila* in laboratory nutrient medium, outgrowth in the IJ receptacle appears to result from periodic increases and decreases in population size (23). Based on these observations, a colonization bottleneck has been proposed in which entry into the receptacle is limited to one or a few cells, or in which cells within the receptacle compete during outgrowth, resulting in a single dominant clonal type (22, 23).

The studies described above relied on destructive sampling from nematode populations: bacteria are extracted by grinding hundreds of IJs, and bacterial CFU are quantified to calculate an average CFU per IJ across the population of nematodes. Alternatively, bacteria are observed within individual nematodes, but only at discrete stages, since the process of sample preparation (e.g., paralysis) and dehydration ultimately leads to nematode death. No studies have yet achieved the direct visualization and quantification of bacterial population dynamics in individual, living host nematodes.

To bridge this methodological gap in the study of host-microbe interactions, we developed a microfluidic system. Microfluidic channels have characteristic dimensions of ~1 to hundreds of micrometers that enable the precise manipulation of small volumes of fluids to create controlled chemical environments (28). Microfluidic systems have been designed for the isolation of individual nematodes and encompass a range of architectures and mechanisms for isolating individual nematodes, including (i) trapping them in droplets of liquid, (ii) isolating them in tapered channels, and (iii) concentrating them in straight channels sealed with valves (29, 30). However, these designs are typically used to study the adult stage of *C. elegans*, which is ~10 to 100 times larger in body size than the dauer larval stage, a developmentally arrested phase similar to IJs of entomopathogenic nematodes (29, 31). These microfluidic devices usually keep whole organisms alive for a relatively short time period—from minutes to hours (32). Much longer time frames—from days to weeks—are required to study the processes underlying the establishment and persistence of long-term microbial symbioses. Thus, tools to study individual dauer or IJ stages of nematodes are not currently available.

This work describes an experimental system for exploring symbiosis between bacteria and nematodes and its application in studying the relationship between *X. nematophila* and *S. carpocapsae*. Individual nematodes are confined within single microfluidic chambers and imaged using optical microscopy; many parallel chambers enable multiple nematodes to be studied simultaneously. The system is simple to operate, does not require chemically induced paralysis (e.g., using levamisole or CO_2_), and eliminates the impact of these reagents and conditions on the population of symbiotic bacteria. We describe the results of using this system to isolate, maintain, and track individual, living nematodes and their microbiota over days and weeks.

## RESULTS

**FIG 2:**
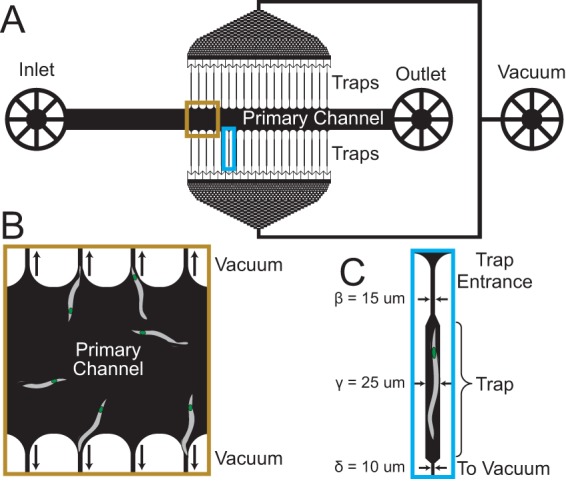
Schematic of microfluidic device for *S. carpocapsae* nematode isolation and maintenance. (A) Device schematic. Nematodes are introduced through the inlet and pushed through the primary channel. Negative pressure applied from the vacuum port pulls nematodes into the traps. (B) Nematodes in the primary channel are pulled into traps with the aid of negative pressure from the vacuum. (C) Physical dimensions of the nematode traps. Note that while the dimensions in the cartoon have been exaggerated for ease of viewing, the labeled dimensions are accurate. For more detail on the trap dimensions, see Table S1.

To investigate *X. nematophila* colonization of and outgrowth in individual IJ receptacles, we fabricated a microfluidic device to isolate and maintain multiple IJ nematodes in individual chambers (referred to as traps) that enabled us to image by microscopy bacteria and nematodes (Fig. 2). We used several criteria in the design of the traps. First, each trap contains an individual IJ. Second, the devices need to maintain IJ viability for weeks to enable long-term observations of the colonization process. Third, the IJs have to be immobilized without the use of chemical paralyzing agents that could impact the colonization process and obscure biological data. Fourth, the system should be simple to operate. The design that we developed to meet these criteria isolates individual nematodes in traps and hydrates them with room-temperature water flowing through the device, except during imaging, when cold water temporarily immobilizes the IJs.

10.1128/mSphere.00530-17.6 TABLE S1  Parameter values in design of microfluidic devices. Download TABLE S1, PDF file, 0.1 MB. Copyright © 2018 Stilwell et al. 2018 Stilwell et al. This content is distributed under the terms of the Creative Commons Attribution 4.0 International license.

### Device design and operation.

We designed and fabricated a microfluidic system in the transparent silicone elastomer poly(dimethylsiloxane) (PDMS) using soft lithography (33). We used a previous design for cell bending experiments (34) as a starting point and revamped it for this study (see Fig. S1 in the supplemental material). The system consists of one inlet (through which nematodes and fluids are introduced) attached to a straight, primary channel that terminates in an outlet (through which excess nematodes and fluids are removed). Traps are connected to each side of the primary channel, are connected to a set of filtering channels (to remove debris and prevent clogging of the traps), and terminate in a second outlet that serves as a vacuum port used to apply negative pressure to the channels and traps (Fig. 2). A description of the specific dimensions of the device is provided in Table S1.

10.1128/mSphere.00530-17.1 FIG S1  Microfluidic device designs. Download FIG S1, EPS file, 8.3 MB. Copyright © 2018 Stilwell et al. 2018 Stilwell et al. This content is distributed under the terms of the Creative Commons Attribution 4.0 International license.

The entire system is 15 by 20 mm long and 3 mm tall and fits on the stage of an inverted microscope. Each of the 38 microfluidic traps was 600 μm long (~1.5 times longer than the average body length of mature IJ nematodes) and 25 μm wide (~1.5 to 2 times wider than the average nematode body [31]) and was designed to hold a single IJ nematode. The width of the traps can be chosen such that the device does not restrict the nematode’s natural sigmoidal movement, and different numbers of traps can be designed into this system for other applications. The height of all channels (including the traps) is 25 μm, and the primary channel is 500 μm wide and 8 mm long (distance from inlet to outlet). The traps had entrance widths ranging from 10 to 25 μm (the body width of IJ nematodes varies with age; this range is for experiments with immature IJs or mature IJs, respectively) (see Table S1). The traps then widened at the region where the nematodes were positioned, such that water could flow around them during experiments. The traps narrowed to 10 μm and were connected to the vacuum port via filtering channels.

We loaded a suspension of isolated IJ nematodes in the inlet of the device using a handheld syringe connected to tubing and attached to the inlet. As we pushed a suspension of the nematodes in water into the primary channel with a handheld syringe, we applied negative pressure using another syringe connected by tubing to the vacuum port that pulled nematodes into the traps. After trapping the nematodes, we relieved the negative pressure and connected the inlet to a syringe through a section of tubing; the syringe was loaded on a syringe pump that perfused the traps with room-temperature water to keep the IJ nematodes hydrated (Fig. 2 and Fig. S5). Since *Steinernema* nematodes are soil dwelling, we also reduced light exposure by covering the system with aluminum foil except while imaging.

### Preparation of colonized IJs and survival in microfluidic traps.

To facilitate direct observation of bacteria within the IJ receptacle using microscopy, we cultivated nematodes colonized with *X. nematophila* cells engineered to express green fluorescent protein (GFP) from a constitutive *lac* promoter. Similar to the processes that occur in an insect cadaver, *S. carpocapsae* nematodes go through reproductive cycles and develop into IJs when cultivated on a lawn of *X. nematophila* cells growing on an agar surface (Fig. 1). Previous research has shown that the *X. nematophila* population exhibits the most drastic measurable changes during the outgrowth process in which a few bacterial cells in newly formed, premigratory immature IJs grow into a population of tens to hundreds of bacterial cells during an IJ maturation process that lasts ~5 days (23). To capture the bacterial population profile during the initiation of colonization (one or a few cells in the IJ receptacle), bacterial outgrowth (one or a few colonized bacteria grow into a population of tens to hundreds of cells), and bacterial persistence in the IJ receptacle (maintenance of a steady bacterial population size as IJs age), we used an SDS treatment (see Materials and Methods) to isolate immature IJs from a mixed population of nematodes from all developmental stages (Fig. 1) (22, 23).

**FIG 3:**
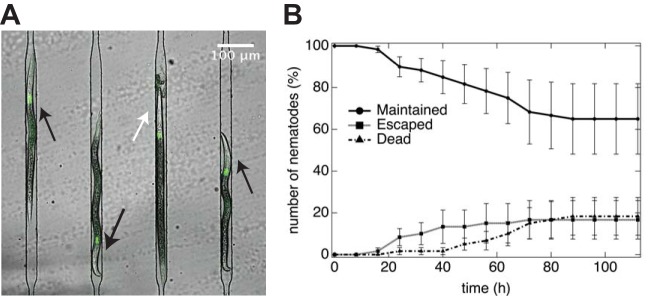
Nematode survival and maintenance. (A) Confocal micrograph of 4 *S. carpocapsae* nematodes isolated in adjacent microfluidic traps. Living nematodes (highlighted with black arrows) display characteristic body curvature and movements associated with healthy nematodes; dead nematodes (highlighted with white arrow) display a straight body posture and disintegration of tissues. *X. nematophila* bacteria constitutively expressing GFP are colonized in the nematode receptacles. (B) Percentage of nematodes in the microfluidic device isolated and alive (circles), escaped (squares), or dead (triangles) over the course of 112 h at room temperature. The average from three independent experiments is indicated; total numbers of nematodes trapped in the device were 18, 22, and 20, respectively. Error bars indicate standard errors.

As the conditions for isolating nematodes and their physical confinement (e.g., restricted space for growth and movement in microfluidic traps) may alter the physiology of IJs, we initially monitored the survival of immature IJs in the device over a 5-day period (Fig. 3). Nematode death is accompanied by a characteristic rigid, straight body posture that lacks movement (Fig. 3A) followed by tissue degradation (35). We searched for this phenotype by optical microscopy and counted the number of viable immature IJs in microfluidic traps during a 5-day period. Out of the total number of *S. carpocapsae* IJs loaded in the traps at the beginning of the experiment (*n* = 60; 3 replicates with 18, 22, and 20 nematodes in each), we found that by the end of the experiment (*t* = 112 h), 60% of the trapped nematodes were viable (*n* = 39), 20% were dead (*n* = 11), and 20% had escaped from the traps (*n* = 10) (Fig. 3B). (A previous study reported that 1 h after SDS treatment of nematodes, the survival rate of *Steinernema feltiae* IJs was 11% to 82% [36], suggesting a reasonable percentage of nematode death caused by SDS treatment followed by physical restriction in our experiments.) Nematodes have a distribution of body sizes, with a subset of IJ nematodes having bodies <10 μm wide that could squeeze through the smallest physical dimensions of the channels and traps and escape. Reducing the trap entrance width reduces the number of nematodes that escape; however, we found that it makes trapping nematodes more difficult, as many nematodes are wider than the entrance dimensions. Consequently, we decided to use our original design and accept the loss of 20% of the nematodes during our experiments.

### Bacterial population dynamics within individual IJs.

**FIG 4:**
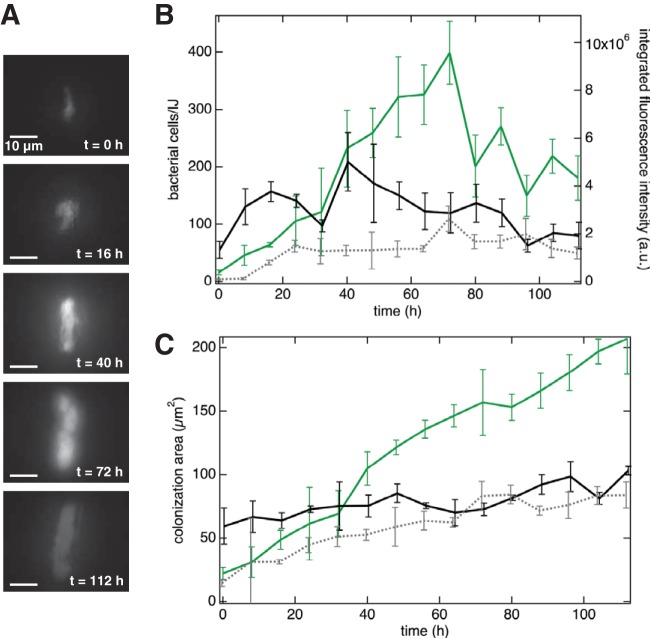
Qualitative and quantitative analysis of bacterial populations in individual, living IJ nematodes. (A) Epifluorescence micrographs of a living, immature IJ nematode receptacle at 0 to 112 h posttrapping in microfluidic device. (B) Bacterial cell numbers and fluorescence intensity as measured in three individual nematode receptacles. Green trace represents data for bacterial cells in panel A, and black and gray traces each represent data collected from individual nematodes. Error bars represent standard deviations of the 9 stepwise, z-stack images. (C) Colonization cross-sectional area in the same nematodes. Error bars represent standard deviations of the 9 stepwise, z-stack images.

In a typical experiment, we simultaneously monitored the population dynamics of *X. nematophila* cells in 10 to 18 live nematodes isolated in parallel microfluidic traps over a 5-day period (Fig. 4). We imaged *X. nematophila* cells in the IJ receptacle, which varies slightly in size among individual nematodes (21 to 33 μm in length and 6 to 9 μm in width of colonized nematodes [37]), using epifluorescence microscopy at 8-h intervals by vertically sectioning the entire structure in 9 steps of 1 μm each, as this approach should allow visualization of the majority of receptacles, regardless of size (Fig. 4A displays micrographs of a representative IJ nematode using this approach). Using a custom IgorPro script, we converted the total integrated fluorescence intensity signal of each section of the receptacle to the approximate number of *X. nematophila* cells by quantifying the integrated GFP intensity across each z-stack and dividing by the fluorescence intensity for a single cell (Fig. 4B). Single-cell fluorescence intensities were determined by analysis of early-time-point images containing few or single bacterial cells using ImageJ (see Fig. S2 for micrographs with isolated bacterial cells). We did not use phase-contrast images for counting single cells, as it is very difficult to distinguish between bacterial cells and nematode cells in these images.

10.1128/mSphere.00530-17.2 FIG S2  Representative images of nematodes colonized with visible single cells. The top four images are from immature IJ nematodes, and the bottom two are from mature IJ nematodes. Single-cell intensity values were determined using ImageJ. Each experimental round was normalized to an average of >10 single-cell intensity values from that experimental round. Download FIG S2, PDF file, 0.7 MB. Copyright © 2018 Stilwell et al. 2018 Stilwell et al. This content is distributed under the terms of the Creative Commons Attribution 4.0 International license.

**FIG 5:**
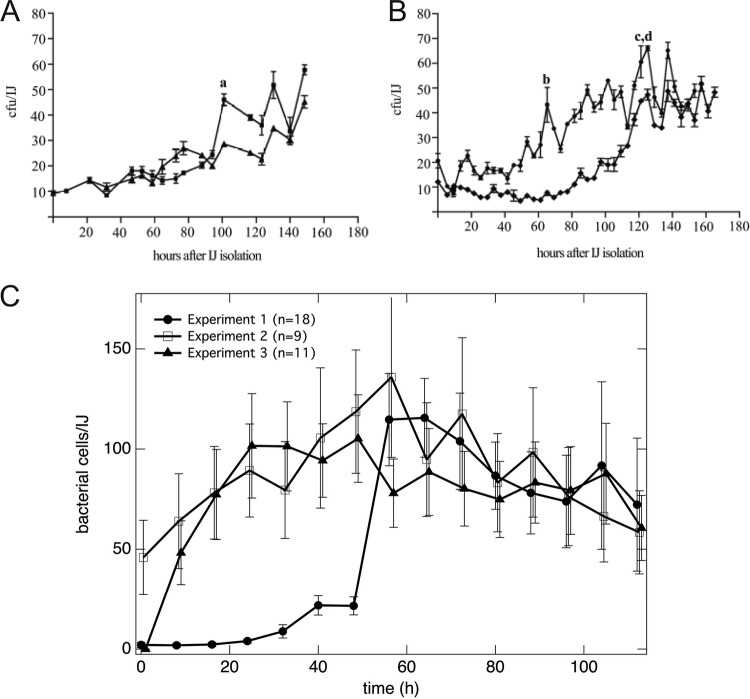
Comparison of traditional grinding experiments with microfluidic device experiments for quantification of *X. nematophila* bacterial population dynamics in *S. carpocapsae* nematodes. (A and B) Traditional grinding experiments include surface sterilization, grinding a subpopulation of nematodes, plating on synthetic medium, and performing bacterial CFU counts. Subpopulations of immature IJs were isolated and assayed every 4 to 8 h. Panels A and B show two replicates each from two separate experiments; each point is the average result for three individual assays. Error bars indicate standard errors of three measurements of the population at each time. Lowercase letters a to d represent time points that were used to calculate maximum growth rate in previous research (23). (C) Analysis of epifluorescence micrographs of GFP-expressing *X. nematophila* in individual *S. carpocapsae* nematodes trapped in a microfluidic device. Each plot represents the average from all living nematodes in the experiment. Error bars indicate standard errors of the population distribution. (Panels A and B are republished from reference 23.)

At the level of individual nematodes, we found that the number of *X. nematophila* cells per unit time fluctuated, such that the number of cells repeatedly increased and decreased in all of the nematodes that we studied (*n* = 50; results for selected nematodes are shown in Fig. 4B). The number of *X. nematophila* cells per unit time varied between individual nematodes. While some nematodes were initially colonized with tens of bacterial cells, others were colonized with a single bacterial cell. To the best of our knowledge, this is the first time that a single bacterium within an IJ receptacle has been observed. The number of colonized bacteria in a single nematode reached a maximum of 743 cells, although no other nematode contained more than 500 bacterial cells at any point. These data are consistent with temporally fluctuating bacterial population sizes observed in past studies based on destructive nematode sampling and ensemble averaging (23) (Fig. 5A and B). In these previously published experiments (presented here as Fig. 5A and B), each data point was assessed by traditional microbiological CFU counting from grinding and plating a subpopulation of nematodes. We compared previous data on bacterial outgrowth (Fig. 5A and B, republished from reference 23) and our data using fluorescence quantification (Fig. 5C). Our direct, fluorescence microscopy measurements quantifying *X. nematophila* cells in *S. carpocapsae* nematodes indicated larger numbers of cells than those revealed by CFU counting, as expected since CFU counts can underestimate cell numbers (36). Note that the error bars in Fig. 5A and B represent measurement errors of the population mean, whereas the error bars in Fig. 5C represent the distribution of the population. We found striking variation in the number of bacterial cells colonizing different IJ nematodes and observed that the number of bacterial cells within a single nematode displayed repeated increases and decreases over time, further supporting the idea that the *X. nematophila* population has periods of growth and death inside the nematode receptacle (see Fig. S3 for bacterial outgrowth dynamics in each IJ) (23).

10.1128/mSphere.00530-17.3 FIG S3  Bacterial cell numbers within an individual IJ nematode over time. For ease of viewing, each plot contains data from no more than five nematodes. Download FIG S3, PDF file, 1 MB. Copyright © 2018 Stilwell et al. 2018 Stilwell et al. This content is distributed under the terms of the Creative Commons Attribution 4.0 International license.

The direct observation of bacterial populations within IJ nematodes enables measurements of both cell number and bacterial colony morphology. We quantified the cross-sectional area of colonization by measuring the area of fluorescence in each IJ receptacle (Fig. 4C). In agreement with previous experiments, we observed the cross-sectional area of colonization displaying an overall increase across the population of nematodes. We also observed that within individual nematodes, the cross-sectional area of colonization fluctuated, similar to the number of bacterial cells that we observed, such that the cross-sectional area increased and decreased over time while displaying an overall increase in area (Fig. 4C). Our direct, fluorescence-based measurements enabled us to extrapolate the bacterial cell density within the IJ receptacle, which occasionally revealed intriguing, unexpected changes: the colonization cross-sectional area increased while the bacterial density decreased (Fig. 4C and S4, green trace). Previous research reported receptacle enlargement correlating with bacterial colonization in *S. carpocapsae* (37), which could affect bacterial density as described above. Our device provides a tool to investigate the dynamics of the nematode host morphology while interacting with bacterial symbionts in future research. Consistently, these data suggest that symbionts within maturing IJ nematodes may experience repeated dissociation and reassociation with each other or with the IVS.

Growth rates of the bacterial population in the nematode receptacle provide an approximate indication of environmental conditions and bacterial metabolism and physiology. To our knowledge, to date there has been only one published study that measured the growth rate of *X. nematophila* in *Steinernema* nematodes, although other studies have measured the growth rate in synthetic media (3, 23, 38). Using an average of individual *X. nematophila* growth curves from each experiment using the microfluidic device and averaging those three independent experiments, we calculated a growth rate of 0.085 ± 0.060 doubling/h (mean ± standard deviation) (see Materials and Methods for growth rate calculations). This value is in good agreement with previous *in vivo* measurements (0.1 doubling/h in the IJ receptacle) and lower than the values measured in synthetic medium (0.62 doubling/h in lysogeny broth) or insect hemolymph (0.41 doubling/h) (3, 23, 38). One advantage of our system over the traditional nematode grinding experiments is that a growth rate can be calculated for the bacteria within each individual nematode and is not limited to a population-based measurement (see Table S2). These data showed nematode-to-nematode differences in symbiont growth rates, with some nematodes showing overall positive symbiont growth (growth rate typically >0 between any three consecutive time points) while other nematodes displayed overall negative growth or death of symbionts (growth rate typically <0 between any three consecutive time points).

10.1128/mSphere.00530-17.7 TABLE S2  Growth rate measurements of *X. nematophila* in individual nematodes. Download TABLE S2, PDF file, 0.1 MB. Copyright © 2018 Stilwell et al. 2018 Stilwell et al. This content is distributed under the terms of the Creative Commons Attribution 4.0 International license.

**FIG 6:**
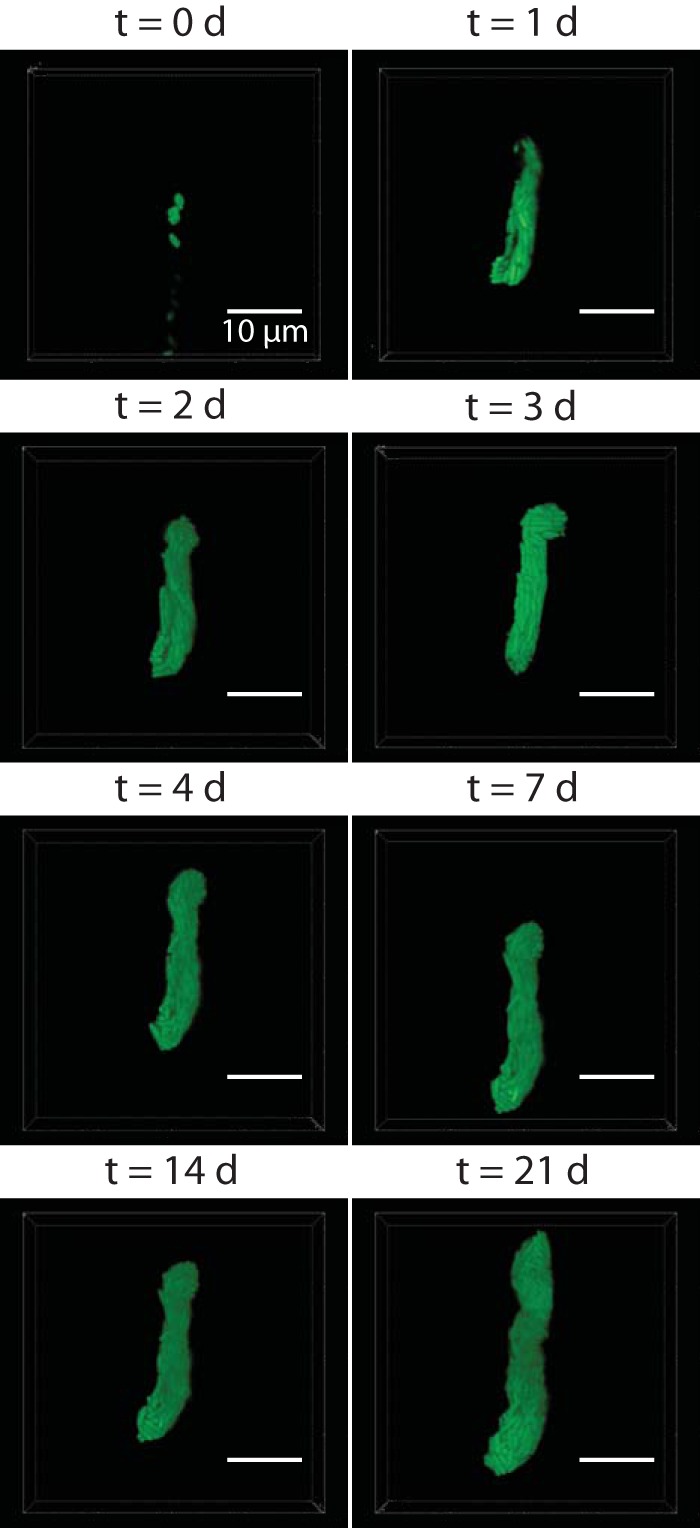
Confocal micrographs of a bacterial population in a receptacle over the course of 3 weeks. An individual living immature IJ nematode (head toward the top) was maintained in the microfluidic device, and the GFP-expressing bacterial population in the receptacle was imaged on days 0, 1, 2, 3, 4, 7, 14, and 21 posttrapping. Bacterial populations were sectioned in 300-nm z-steps.

We next applied our microfluidic device to study the bacterial colonization of nematode hosts at single-cell resolution. For these experiments, we reduced the widths and heights of the microfluidic traps to ~10 μm to further restrict the movement of nematodes, enabling us to perform confocal microscopy on *X. nematophila* cells and recreate the topology of the community in three dimensions (3D). As in the epifluorescence measurements, we cooled the nematodes from room temperature to 4°C for 30 min before imaging to further reduce nematode movements while shielding them from ambient light. Using this method, we imaged bacterial populations at single-cell resolution in the nematode receptacle over 21 days. We optically “sectioned” the bacterial population in the receptacle in 300-nm steps once per day for the first 5 days and once a week thereafter. Figure 6 shows a 3D reconstruction of a representative bacterial population within a nematode receptacle over time. 3D reconstructions of the confocal micrographs revealed bacterial population topology changes in the IJ receptacle over weeks. For instance, between days 2 and 3, we observed a portion of the bacterial colony grow an offshoot (Fig. 6, day 3, top part of colony) that disappeared the following day. Intriguingly, after the initial outgrowth period, the bacterial population was steady until day 14, after which the population underwent another period of growth. Future experiments for longer durations and with increased temporal resolution will reveal more information on the dynamics of persistence in the IJ nematode.

## DISCUSSION

In this report, we describe a microfluidic system that enables fundamental questions of host-microbe interactions to be addressed at the level of individual nematode hosts and individual bacterial cells. The data that we acquired using this method are generally consistent with those published using traditional, culture-based microbiological methods and yet expand on earlier observations (23). First, by tracking individual nematodes we confirmed the previously established model that one or very few “founder cells” initiate IJ colonization (23). Our microfluidic data made possible the novel observation of IJs with only one bacterial cell within the receptacle, which adds strong support to the founder cell model of colonization initiation. Second, our data demonstrate increases and decreases of a population of *X. nematophila* cells in a single nematode over time. Due to technical caveats, previously observed fluctuations in apparent bacterial population size could be explained by sample-to-sample variation in nematode or bacterial physiology. Here, we were able to directly observe bacterial growth and death within a single nematode, supporting the robust conclusion that bacterial populations within IJs are dynamic. Third, by combining microfluidic traps with confocal microscopy, we imaged individual living nematodes and their symbionts for >3 weeks and reconstructed the structure of the bacterial population over time. These experiments confirmed our previous data demonstrating dynamics in the bacterial population topology in the first few days of bacterial outgrowth while also revealing bacterial population dynamics weeks after colonization. These experiments demonstrate a unique new capability for pushing the boundary of host-microbe interaction studies to a new level of resolution.

The sharp and repeated increases and decreases in the symbiont population of every nematode in our study are an intriguing phenomenon. We propose several hypothetical, non-mutually exclusive models to explain periodic changes in the symbiont population. In model 1, physiological changes, e.g., internal patterns created by a circadian rhythm or the nematode’s physical movement, trigger nutrient influx to the receptacle that affects bacterial metabolism. Rhythmic physiological changes in other organisms have been shown to affect bacterial symbionts (39); however, this phenomenon has not yet been studied in *S. carpocapsae* nematodes. Other physiological changes, such as nematode movement, may stimulate peristalsis in the gut; facilitate mixing, mass transport, and diffusion within this organ; and alter bacterial growth and structures in the receptacle. It is unknown whether the nematode host continuously provides nutrients for the bacteria during long-term colonization. This microfluidic device facilitates future investigations into the nutritional state of the receptacle in individual nematodes.

In model 2, it is also possible that fluctuating populations sizes during outgrowth result from bacterial cell death and subsequent competition for the utilization of the nutrients released from dead cells. In this model, subpopulations of bacteria that are adapted to utilizing released nutrients would have a growth advantage within the nematode receptacle and become the dominant clone, in a process similar to the growth-advantage-in-stationary-phase (GASP) phenomenon that has been reported for *Escherichia coli* (40). This type of competition could give rise to the observed mono- or biclonal symbiont population within IJs, which sector within the receptacle (23). Using the microfluidic device, we observed that in the initiation stage of immature IJ colonization, individual nematodes associated with 2 to 5 spatially separated individual cells or clusters (see Fig. S2 in the supplemental material), which may be indicative of discrete clonal populations competing with each other for nutrients. Future implementation of the microfluidic system using wild type, metabolic mutants, and/or strains of *X. nematophila* expressing different colors of fluorescent proteins will enable direct visualization of the nutritional environment and competition over long timescales and will contribute significantly to our knowledge of the critical transmission stage of the *Steinernema-Xenorhabdus* complex.

Our third model entails population growth and death arising due to phenotypic switching. *X. nematophila* exhibits a phenotypic variation phenomenon termed virulence modulation, in which cells switch between mutualistic and pathogenic states (41–43). The IJ transmits *X. nematophila* from a niche in which it expresses mutualistic behaviors (the insect cadaver in which it supports nematode reproduction) to one in which it expresses pathogenic behaviors (the blood cavity of a newly infected living insect host) (Fig. 1). It is hypothesized that the IJ environment selects for or induces symbiont switching from the mutualistic to the pathogenic state to preadapt bacterial cells for the upcoming infection stage (44). It is possible that repeated growth and death of the bacteria in IJs reflect the process by which the mutualistic-to-pathogenic phenotypic switch is occurring. For example, population decline may be caused by selective death of cells in the “mutualistic state,” followed by division of cells in the “pathogenic state.” The development of tools for monitoring bacteria within individual nematode hosts opens the door to observing such dynamics in real time, for example by examining nematodes colonized by *X. nematophila* expressing state-specific fluorescence reporters.

Error contributes to our measurements of bacterial population size. For instance, while imaging, some nematodes move in response to light, which can interfere with fluorescence measurements. Traps with smaller dimensions than those used here can constrain nematode movement and reduce this source of error. Epifluorescence microscopy collects light from focal planes above and below the sample that can introduce uncertainty into fluorescence quantification. This effect can be transcended by confocal microscopy; however, analyzing confocal data is often nontrivial, as bacterial cells are aligned in all directions and cell outlines are not always easily distinguishable, which can introduce bias and uncertainty when counting cells, in addition to the analysis being less easily automated. The wide availability of epifluorescence microscopy in part led us to select this method for this first study. Another potential source of error may arise from normalizing integrated intensity to single-cell intensity, as fluorescence will vary from cell to cell (45). Nematode-to-nematode differences in initial bacterial load coupled with the current inability to synchronize the nematodes through the reproductive cycle will also increase the heterogeneity in measurements.

Several microfluidic devices that isolate individual nematodes have been published; however, the majority of these systems require microfluidics expertise for their operation (29, 30). Our goal was to provide the field with a tool to enable microbiology and nematology laboratories to perform long-timescale, single-nematode studies. Consequently, the design minimized the number of accouterments required to operate the systems: disposable syringes, needles, and tubing. We provide a link to download a vector graphics file of the device blueprint in the supplemental material; using this file, laboratories can purchase a device “master” and fabricate their own microfluidic devices (see the supplemental material for device design downloads). The device is easy to operate, and we anticipate that experts in biology areas outside engineering will find the devices valuable in their studies of single organisms interacting with symbionts.

The device outlined in this work overcomes several limitations of previous techniques. First, the device surmounts three key limitations of the CFU-counting approach. (i) Combining different aliquots of a nematode population at each time point is likely to increase variability in the number of CFU measured per IJ, since both the number of nematodes extracted for the sample and the colony growth conditions vary. (ii) Culture-based methods underestimate cell counts, as they do not accurately represent nonculturable or slow-growing cells (46). (iii) The CFU-counting approach is a population-based measurement, and measurements of many nematodes simultaneously will dilute any dynamics within a single nematode. As such, variations in CFU counts are heavily masked by experimental error, and these counts may miss subtle changes in bacterial populations within a single nematode, which is an important aspect of the symbiosis (13). Second, using microscopy, researchers have examined nematodes containing bacteria expressing a fluorescent protein and categorized the colonization by the fraction of the receptacle filled. While valuable, this technique is highly subjective, as the fraction of receptacle occupied by bacteria is not measured but rather is estimated relative to other nematodes in the sample. Our device enables a quantitative analysis of the cross-sectional area of colonization while approximating the number of bacterial cells in the receptacle, which is unachievable with traditional techniques. This feature facilitated the observation of the occasional symbiont population increasing in colonization area while decreasing in approximate cell numbers (Fig. 4, green trace) and has potential for investigating the relationship between host receptacle morphology and symbiont density. Third, our device enables accurate growth rate measurements of bacterial populations within individual nematodes that open the door for exploring new areas of symbiosis, e.g., investigating whether positive or negative symbiont growth in individual nematodes is correlated with temperature, nematode gene expression, pheromone or small-molecule production, or particular behaviors such as IJ dispersal.

We envision several ways that this device can be improved upon or altered for future experiments and different studies. Temperature gradients could be introduced into the device such that different traps within the device operate at different temperatures, potentially influencing the development of the symbiotic relationship. Pressure-driven valves could be added to the device to aid in nematode trapping, potentially increasing the percentage of nematodes that remain trapped in the device during experiments (47). Alterations of the trap dimensions will enable the isolation of other organisms with a range of sizes for long-term studies (30).

Microfluidics provides one approach to immobilize and maintain individual nematodes and has been successfully applied to neuroscience and behavioral studies of *C. elegans* (48, 49). In *C. elegans*, population-based studies show that the nematode entry and development of the dauer stage involve significant changes in the expression of genes of hormonal and metabolic regulation that control the longevity and behaviors of the animals (50, 51). Recent research has begun to reveal that bacteria, a food source for *C. elegans*, influence the nematode dauer formation and longevity (52), stimulating questions about bacterium-nematode interactions and host responses using dauer-stage *C. elegans* as an emerging model of symbiosis. The microfluidic platform that we developed in this study provides a possible solution to track individual *C. elegans* dauer organisms over long periods of time to investigate the nematode physiological changes. In *Steinernema* nematodes, the IJ stage provides a valuable opportunity to study intriguing questions regarding symbiont and host physiology that currently are not known to occur in *C. elegans*. Here, we present a new microfluidic platform and design that are compatible with the constraints of loading, maintaining, and immobilizing IJ *Steinernema* nematodes and imaging their symbiotic bacteria for long-term time course experiments. The design also serves as a template for more complicated experiments or experiments with other organisms, which may facilitate the study of microbes that cannot be grown with traditional microbiology methods, and for studying microbiomes. The colonization, survival, growth, and persistence of microbial symbionts in host animals are central to the health and function of these organisms, the details of which will be unraveled with the aid of new tools, such as the microfluidic system that we describe in this paper.

## MATERIALS AND METHODS

### Bacterial strain construction and growth.

We created the GFP-expressing *X. nematophila* strain HGB 2110 by inserting *gfp* in plasmid pURR25 (mini-Tn*7*-KS-GFP) (26, 53) from the *Escherichia coli* donor strain (HGB 1262) into the attTn*7* site in the genome of the recipient *Xenorhabdus nematophila* wild-type bacterial strain (HGB 1969) using triparental conjugation with pUX-BF13 (HGB 283) as a helper plasmid. The site-specific insertion at attTn*7* was confirmed by antibiotic resistance, sensitivity, and PCR amplification using primers mTn*7*-befKanR (GTCGACTGCAGGCCAACCAGATAAGT) and AttTn*7*-ext (TGTTGGTTTCACATCC), yielding positive a band of ~500 bp.

We streaked bacteria on LB agar supplemented with 1 g/liter sodium pyruvate (54). Overnight cultures were grown in LB liquid medium incubated at 30°C with rotation on a cell roller. Agar or liquid medium was supplemented with the appropriate concentrations of antibiotics: 50 μg/ml kanamycin and 30 μg/ml chloramphenicol for *E. coli* or 15 μg/ml chloramphenicol for *X. nematophila*. We incubated bacteria growing in liquid medium or on agar infused with liquid medium at 30°C in the dark.

### Nematode propagation and aposymbiotic IJ preparation.

*S. carpocapsae* nematodes were propagated through *Galleria mellonella* insect larvae (Grubco) and stored in water at room temperature. Conventional nematodes produced from three independent rounds of propagation were used to prepare independent batches of axenic eggs and aposymbiotic nematodes (nematodes do not carry bacterial symbionts in the receptacle) (26), each used in one independent experiment.

### Nematode colonization assay and immature IJ isolation.

We grew bacterial lawns by plating 600 μl of an overnight culture of *X. nematophila* onto lipid agar (55) and incubated the culture at 25°C for 48 h in the dark. For each replicate, 5,000 aposymbiotic IJs (500 μl of 10 IJs/μl in LB medium) were surface sterilized, added to the bacterial lawn, and incubated at 25°C in the dark. Six days later, we sampled nematodes from the bacterial lawn and examined them by microscopy to confirm the formation of immature IJs. Immature IJs were isolated by adding sterile, deionized water to the lipid agar plates to suspend the nematodes. The nematodes were allowed to settle, after which the supernatant was removed and the nematodes were resuspended in a 1% SDS solution (in water), followed by 20 min of shaking (23). The SDS solution kills nematodes in other developmental stages except for IJs, which we centrifuged for 10 min at 3,000 rpm. We then discarded the supernatant and resuspended the nematodes in sterile water. After isolation and washing of immature IJs, we performed a modified surface-sterilization protocol (55) by treating samples with 0.5% bleach for 2 min. The suspension was then filtered through a filter, followed by 3 washes with sterile water using vacuum aspiration. This bleach treatment removed cadavers of dead nematodes (mostly non-IJs) in the sample, leaving us with isolated IJ nematodes.

### Microfluidic device fabrication and operation.

We fabricated microfluidic device masters using standard soft lithography techniques (33). Briefly, we created masters by transferring a pattern from a computer-aided design (CAD) computer file into SU-8 3025 photoresist (Microchem, Newton, MA) on silicon wafers using photolithography. We used a benchtop spin coater (Laurell Technologies Corp., New Wales, PA) to deposit a thin layer of SU-8 3025 onto a clean wafer at 3,000 rpm for 30 s, followed by a postexposure bake step and UV exposure to transfer the pattern into photoresist. To transfer the pattern into SU-8 3025, we used negative photomasks (CAD/Art Services Inc., Bandon, OR) and a custom aligner and UV light source. Excess photoresist was removed using SU-8 developer (Microchem, Newton, MA). Microfluidic channels had a height of 25 μm, and microfluidic elements had the dimensions described in this work and outlined in Table S1 in the supplemental material. The width of the primary channel was 500 μm, and the distance between the inlet and outlet was 8 mm. The device contained 38 traps. The traps had variable entrance widths, ranging from 10 to 25 μm. The traps then widened to 10 to 25 μm with a length of 600 μm. The traps then narrowed to 10 μm and connected to the vacuum port via a set of filtering channels. We silanized masters with a vapor of (tridecafluoro-1,1,2,2-tetrahydrooctyl)trichlorosilane (Gelest Inc., Morristown, NJ) to facilitate removal of cured layers of poly(dimethylsiloxane) (PDMS) from the master. We cast PDMS (10:1 ratio of base to cross-linking agent [Sylgard 184; Dow Corning, Midland, MI]) on the masters to a depth of ~3 mm and then cured the polymer at 100°C for >2 h. We peeled cured PDMS layers embossed with microfluidic designs from the master and trimmed them with a razor to a suitable size for bonding to a glass slide. A 1-mm-diameter tissue bore was used to punch inlets and outlets in the PDMS device. We cleaned the surface of PDMS devices with frosted office tape, immersed the PDMS layers in a container with acetone placed in a sonicating water bath for 20 to 30 min, and then dried them using compressed air. Glass coverslips and clean, dry PDMS devices were exposed to oxygen plasma for 1 min and pressed into conformal contact to bond the glass and PDMS. Devices were put into a 100°C oven for >30 min to ensure efficient bonding. To create the connections to the microfluidic device, we trimmed 19-gauge needles to ~1-cm pieces and blunted the ends. These connectors were inserted into Tygon microbore polyvinyl chloride (PVC) tubing (0.030-in. inside diameter [i.d.], 0.090-in. outside diameter [o.d.], 0.030-in. wall), which had been cut to a length amenable to our workspace, and inserted into the PDMS device via the inlet, outlet, and vacuum port. The other end of the tubing was connected to a blunted 19-gauge needle attached to a syringe (for the introduction of nematodes, hydration, and vacuum) or inserted into a waste beaker (for the outlet) (see Fig. S5 for representation of experimental setup).

We diluted nematodes to a concentration of <10 nematodes per μl and introduced them into the device with a 1-ml Luer lock syringe. Simultaneously, we applied a vacuum to pull nematodes into the chamber with a 5-ml Luer lock syringe. Once nematodes were loaded, we used a syringe pump to make water flow into the device at a rate of 1,000 μl/h to maintain the hydration of the nematodes.

### Symbiotic bacterium imaging *in vivo.*

All epifluorescence images were acquired using an Eclipse Ti inverted microscope (Nikon, Tokyo, Japan) equipped with a CoolSNAP HQ^2^ camera (Photometrics, Munich, Germany). Images were taken using a Nikon S Plan Fluor extra-long working distance (ELWD) 40× objective. Each nematode was imaged with 9 steps of 1 μm each to construct a z-stack to capture the depth of the receptacle. All confocal images were taken using a Nikon A1R-Si+ confocal microscope (Nikon, Tokyo, Japan) equipped with high-sensitivity GaAsP detectors. Nematodes were sampled in 300-nm z-steps.

### Data acquisition and analysis.

Quantitative analysis of fluorescently labeled bacterial cells was performed using a custom script in IgorPro. Briefly, cells were detected using the ImageThreshold function, and then a mask was created in the area of detected cells. The pixel intensities were combined to produce an integrated fluorescence intensity. The number of pixels was counted and converted to an area measurement based on a pixel-to-micrometer conversion. The integrated fluorescence intensity was converted to an approximate bacterial cell number by dividing the integrated intensity by the integrated intensity of a single cell. Single-cell images were collected at the beginning of experiments and analyzed in ImageJ.

### Calculating growth rate.

In order to calculate the bacterial growth rate, we used the following equation: *k *= [log_2_(*n*_2_/*n*_1_)]/(*t*_2_ − *t*_1_), where *k* represents the growth rate in doublings per hour and *n*_*x*_ represents the number of bacterial cells at time *x*, represented by *t*_*x*_.

10.1128/mSphere.00530-17.4 FIG S4  Quantitative analysis of epifluorescence micrographs of GFP-expressing *X. nematophila* in individual *S. carpocapsae* nematodes trapped in a microfluidic device. This plot shows bacterial cell density over time in the same three nematodes as shown in Fig. 4. The green trace represents cells from Fig. 4A. Bacterial cell density is calculated by dividing the number of bacterial cells by the cross-sectional area of colonization. Download FIG S4, PDF file, 1.5 MB. Copyright © 2018 Stilwell et al. 2018 Stilwell et al. This content is distributed under the terms of the Creative Commons Attribution 4.0 International license.

10.1128/mSphere.00530-17.5 FIG S5  Cartoon representation of device assembly. A syringe pump pushes water out of a syringe, through a piece of tubing, and into the microfluidic device for nematode hydration. Water exits the device through another piece of tubing and is collected in a waste beaker. Negative pressure is applied via a handheld syringe connected to the device through tubing to pull nematodes into the microfluidic traps. Download FIG S5, PDF file, 0.4 MB. Copyright © 2018 Stilwell et al. 2018 Stilwell et al. This content is distributed under the terms of the Creative Commons Attribution 4.0 International license.
