# Supplementary material for: Studying the Symbiotic Bacterium Xenorhabdus nematophila in Individual, Living Steinernema carpocapsae Nematodes Using Microfluidic Systems
Source: mSphere. 2018 Jan 3;3(1):e00530-17. doi: 10.1128/mSphere.00530-17 (PMC5750387; doi:10.1128/mSphere.00530-17)
Supplement: TABLE S1 [file sph001182439st1.pdf]

Table S1. Parameter values in design of microfluidic devices

		Epifluorescence[b]		Confocal[c]			
Parameter[a]	Description	E1 (μm)	E2 (μm)	C1 (μm)	C2 (μm)	C3 (μm)	C4 (μm)
α	Entrance radius	75	75	75	75	75	75
β	Connection (w/L)	15/200	10/200	10/200	10/200	10/200	15/200
γ	Trap (w/L)	25/600	25/600	25/600	10/600	15/600	20/600

[a]Various combinations of parameter α, β, and γ values (see Fig. 2C for details) are used for designs of microfluidic devices to fit various sizes of nematodes in either epifluorescence microscopy or confocal microscopy experiments. Parameter α = radius of the circular arc used for the nematode trap entrance; Parameter β = dimensions of the connection between trap entrance and trap (shown in width/length); Parameter γ = dimension of the trap (shown in width/length).

[b]The combination of parameter values used for the device designs in epifluorescence microscopy experiments (E1= epifluorescence design 1 and E2= epifluorescence design 2) are listed.

[c]The combination of parameter values used for the device designs in confocal microscopy experiments (C1-C4= Confocal design 1-4) are listed.
