# Supplementary figures and images for: Studying the Symbiotic Bacterium Xenorhabdus nematophila in Individual, Living Steinernema carpocapsae Nematodes Using Microfluidic Systems
Source: mSphere. 2018 Jan 3;3(1):e00530-17. doi: 10.1128/mSphere.00530-17 (PMC5750387; doi:10.1128/mSphere.00530-17)

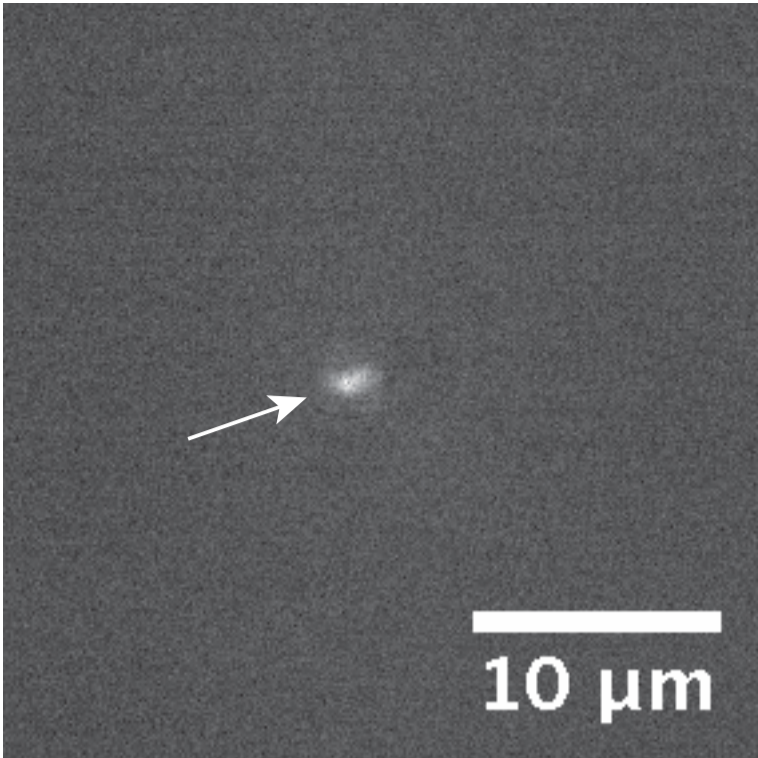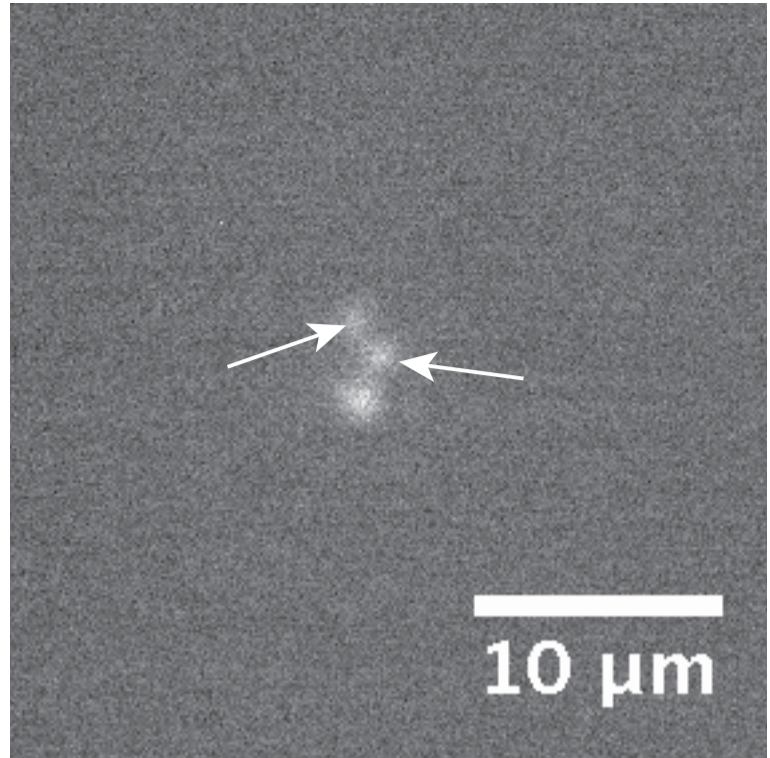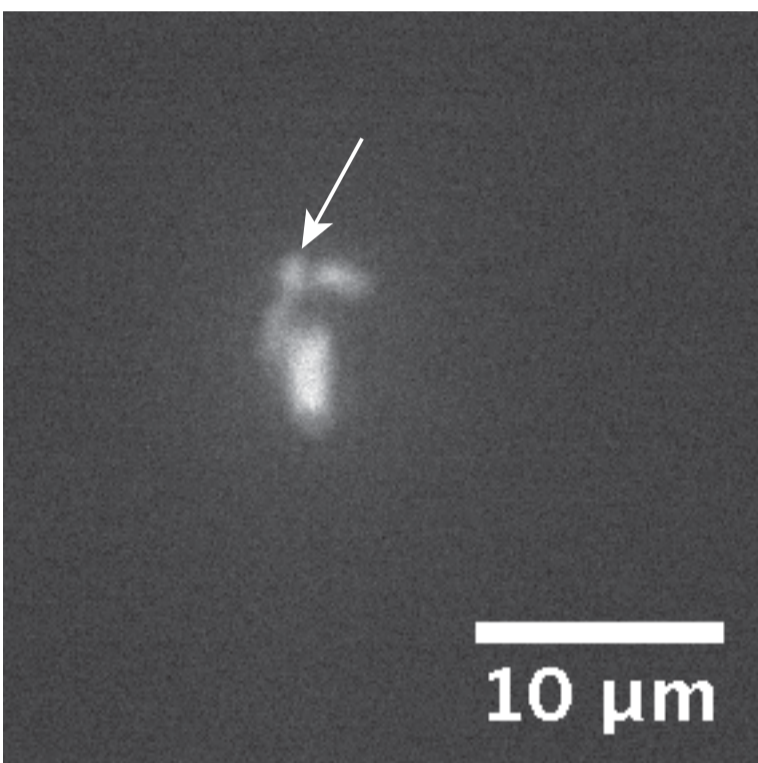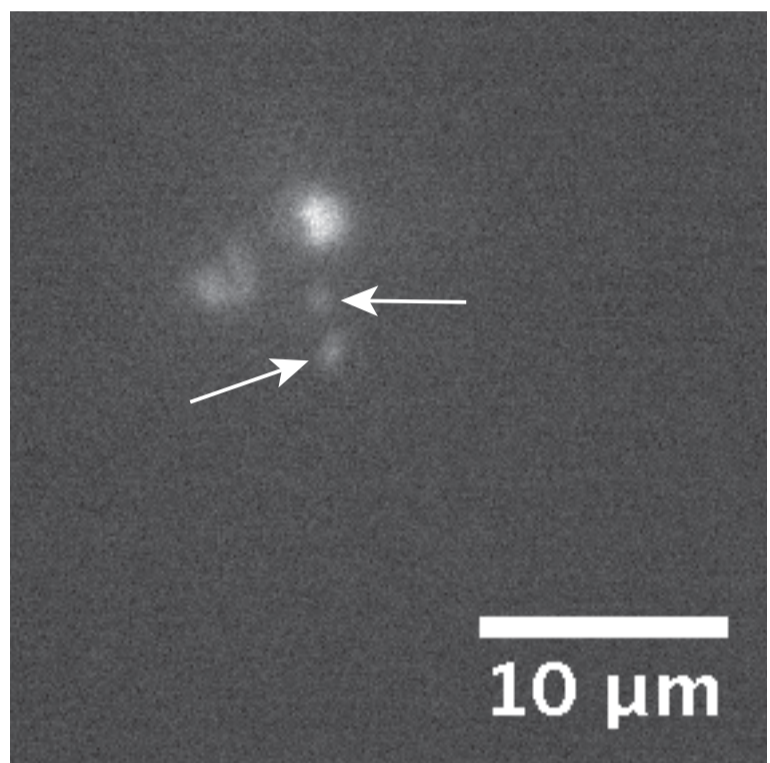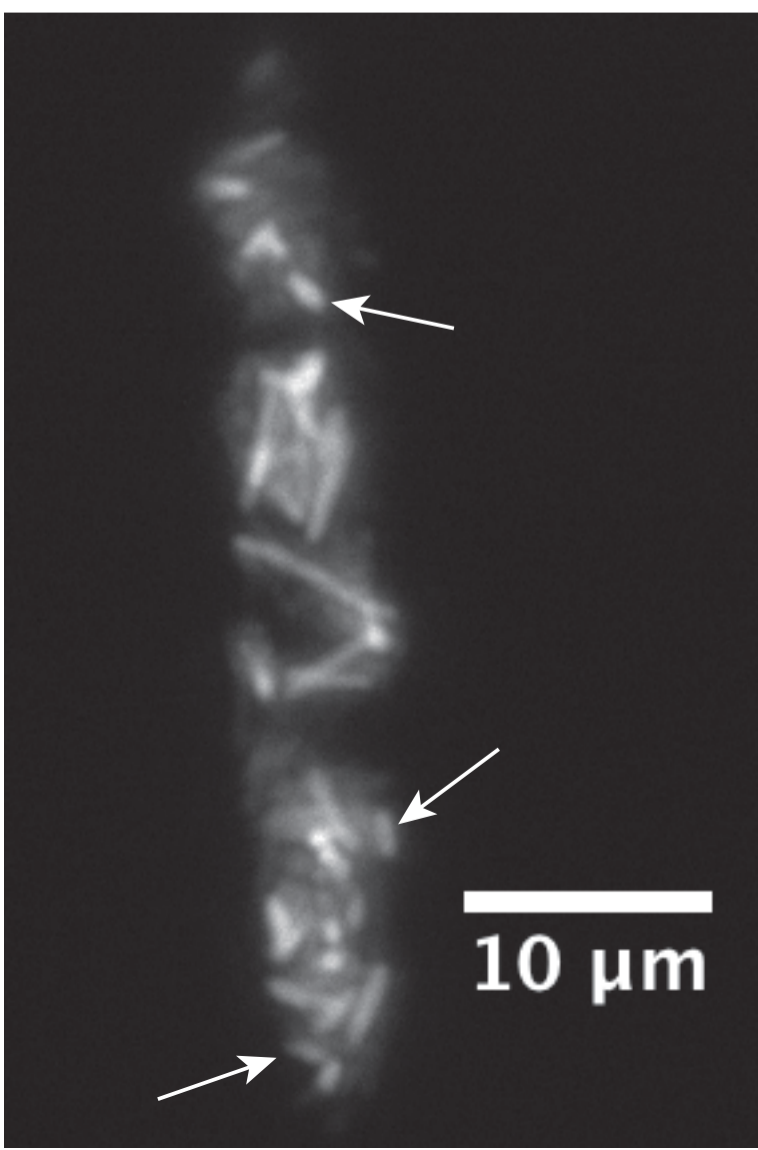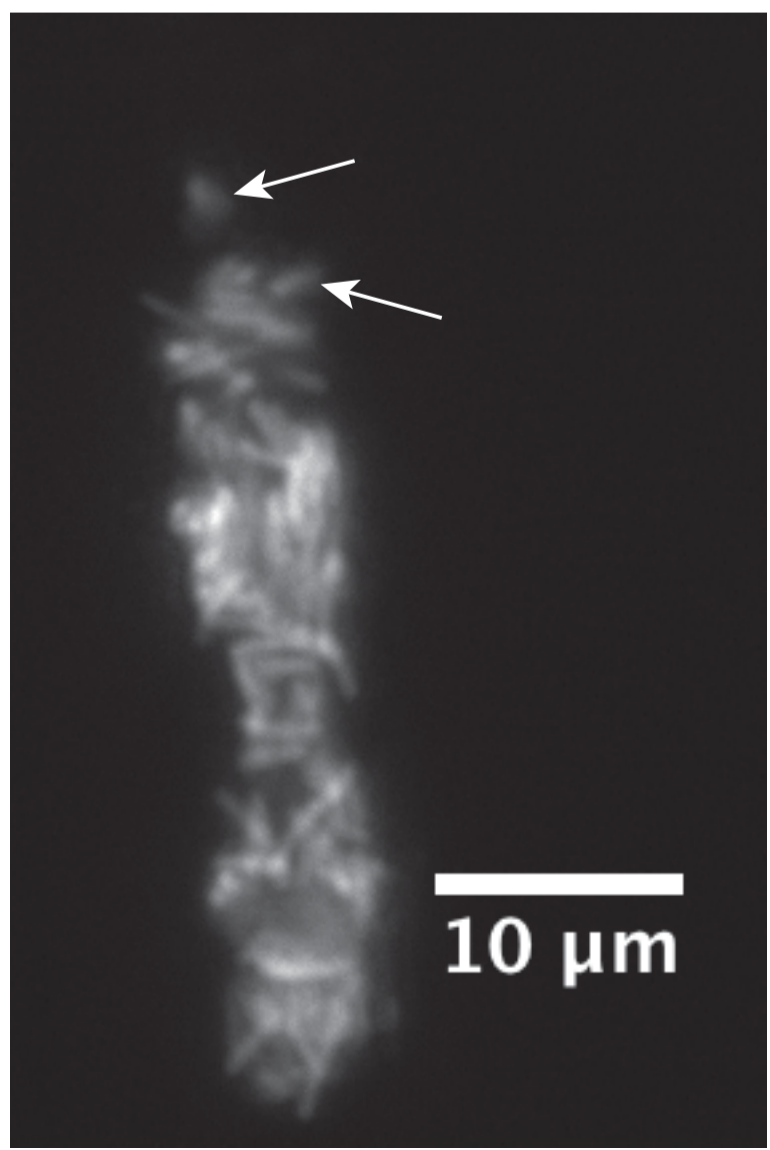

Supplement: FIG S2 [file sph001182439sf2.pdf]

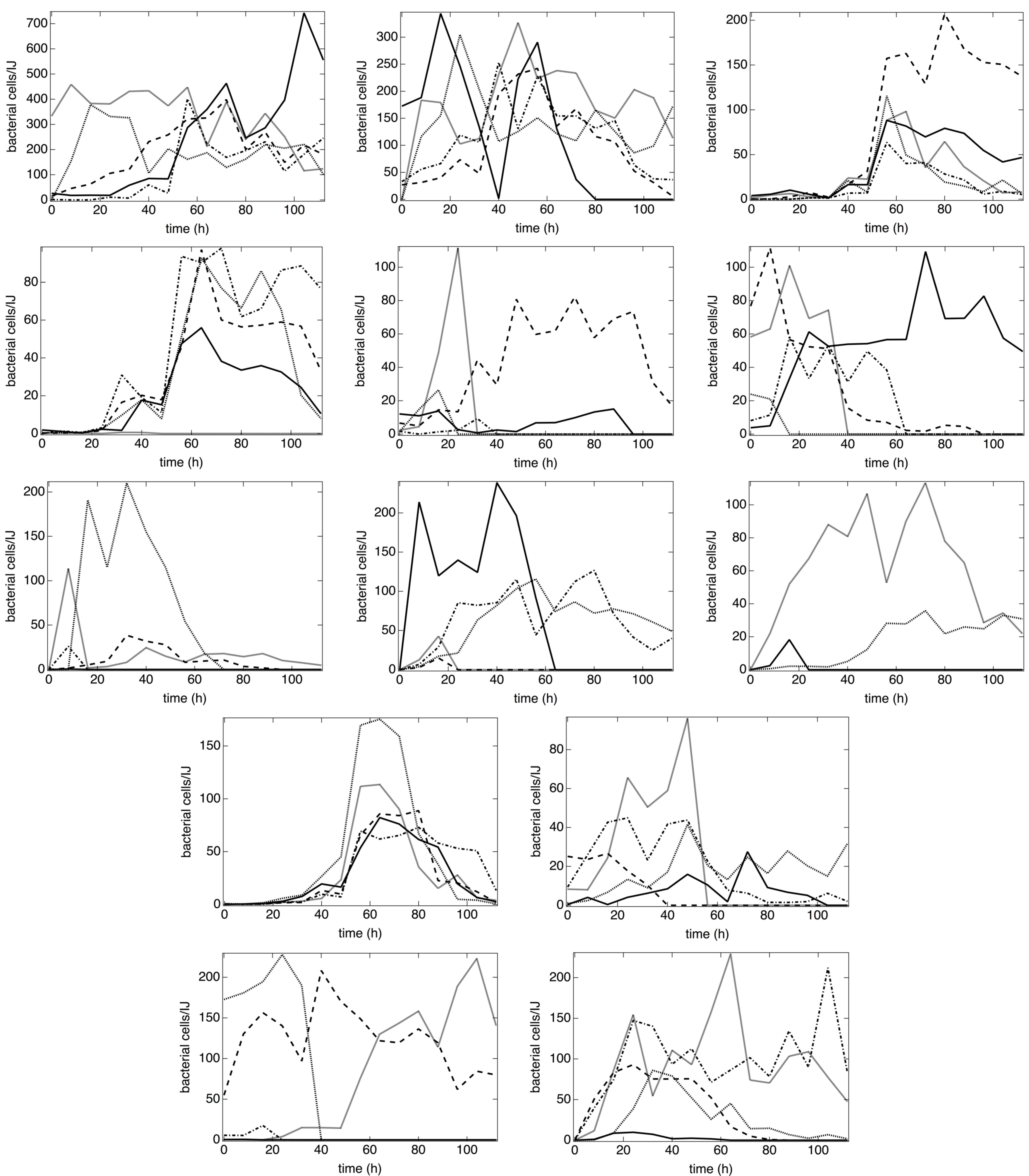

Supplement: FIG S3 [file sph001182439sf3.pdf]

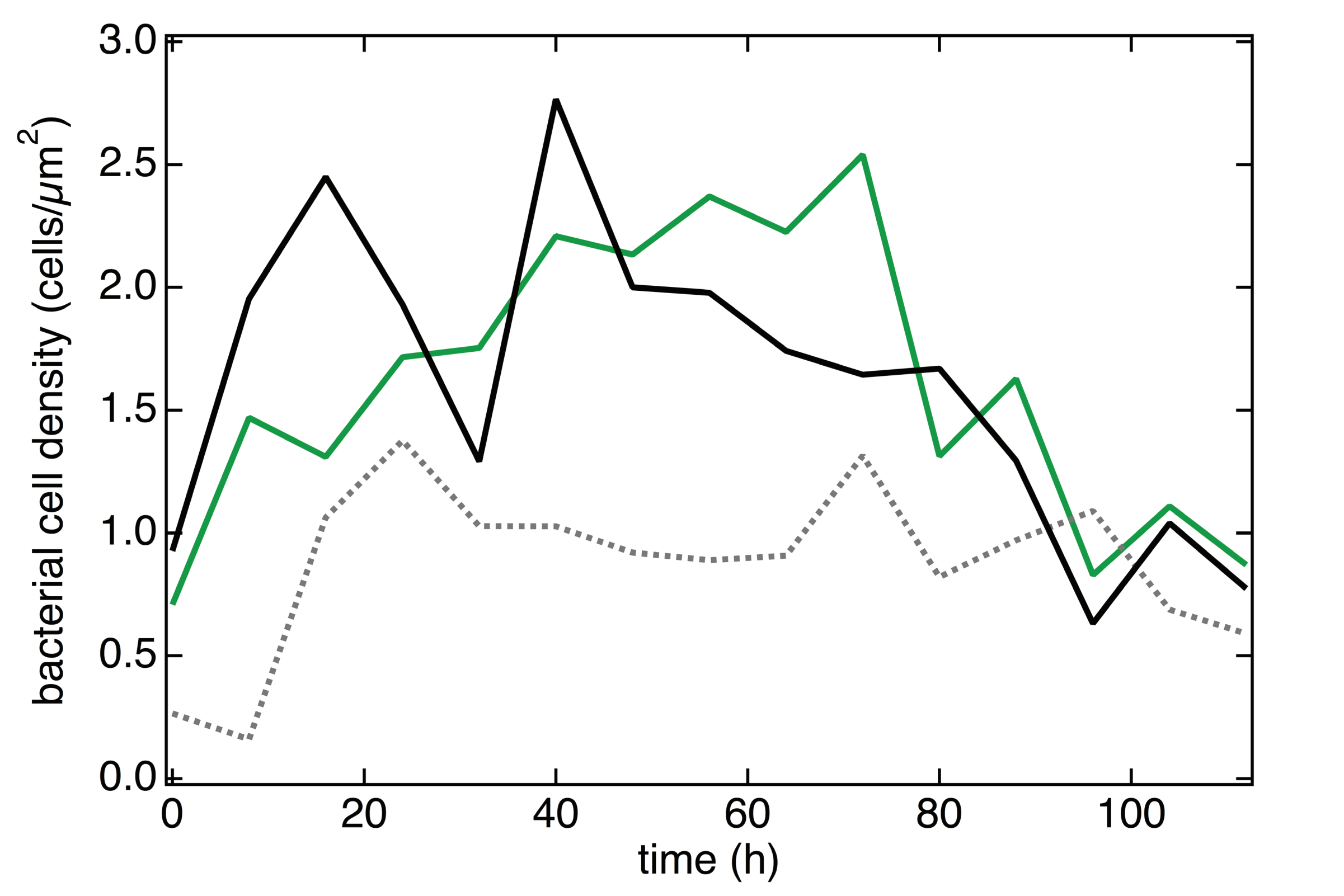

Supplement: FIG S4 [file sph001182439sf4.pdf]

# Syringe Pump

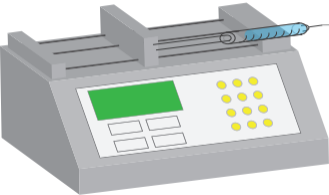

# Waste

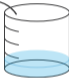

# Microfluidic Device

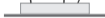

# Vacuum

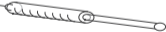

Supplement: FIG S5 [file sph001182439sf5.pdf]
