# Supplementary material for: Studying the Symbiotic Bacterium Xenorhabdus nematophila in Individual, Living Steinernema carpocapsae Nematodes Using Microfluidic Systems
Source: mSphere. 2018 Jan 3;3(1):e00530-17. doi: 10.1128/mSphere.00530-17 (PMC5750387; doi:10.1128/mSphere.00530-17)
Supplement: TABLE S2 [file sph001182439st2.pdf]

Table S2. Growth rate measurements of *X. nematophila* in individual nematodes

Experiment Number	Maximum Growth Rate (doublings/h)	Average Growth Rate (doublings/h) (mean ± st. dev.)
1	0.21	0.015 ± 0.081
1	0.22	0.0098 ± 0.090
1	0.32	0.031 ± 0.13
1	0.24	0.019 ± 0.11
1	0.20	0.018 ± 0.091
1	0.18	0.039 ± 0.078
1	0.34	0.035 ± 0.11
1	0.16	0.018 ± 0.087
1	0.14	-0.0019 ± 0.1
1	0.2	0.023 ± 0.090
1	0.17	0.0087 ± 0.097
1	0.21	0.011 ± 0.092
1	0.42	0.015 ± 0.15
1	0.29	0.0073 ± 0.18
1	0.11	0.020 ± 0.047
1	0.17	0.019 ± 0.081
1	0.33	0.050 ± 0.12
1	0.21	0.031 ± 0.069
1	0.15	-0.059 ± 0.21
1	0.37	0.036 ± 0.13
2	0.014	0.0021 ± 0.013
2	0.10	0.052 ± 0.075
2	0.096	0.016 ± 0.041
2	0.078	0.0024 ± 0.037
2	0.14	0.0018 ± 0.083
2	0.22	0.12 ± 0.086
2	0.079	0.00064 ± 0.046
2	0.21	0.13 ± 0.11
2	0.054	-0.0064 ± 0.036
2	0.13	0.086 ± 0.056
2	0.11	0.0060 ± 0.057
2	0.17	0.016 ± 0.053
2	0.042	0.0055 ± 0.031
2	0.15	0.020 ± 0.069
2	0.10	-0.023 ± 0.059
2	0.24	0.020 ± 0.15
2	0.10	0.037 ± 0.050
2	0.092	0.023 ± 0.057
2	0.13	-0.01 ± 0.065
2	0.11	-0.0098 ± 0.072
2	0.45	-0.015 ± 0.22
3	0.18	0.0058 ± 0.10
3	0.18	0.0098 ± 0.074
3	0.065	-0.0033 ± 0.033

3	0.081	-0.0029 ± 0.048
3	0.28	0.010 ± 0.11
3	0.077	0.0051 ± 0.048
3	0.044	-0.042 ± 0.069
3	0.10	-0.022 ± 0.12
3	0.054	-0.035 ± 0.051
3	0.13	0.0011 ± 0.080
3	0.059	-0.013 ± 0.046
3	0.14	0.018 ± 0.049
3	0.13	0.012 ± 0.062
3	0.061	0.0027 ± 0.036
3	0.078	-.000023 ± 0.044
3	0.11	0.027 ± 0.048
